# Salvage Ultrasound-Guided Robot-Assisted Video-Endoscopic Inguinal Lymphadenectomy (RAVEIL) as a Metastasis-Directed Therapy (MDT) in Oligoprogressive Metastatic Castration-Resistant Prostate Cancer (mCRPC): A Case Report and Review of the Literature

**DOI:** 10.3390/curroncol32020115

**Published:** 2025-02-18

**Authors:** Rafał B. Drobot, Marcin Lipa, Artur A. Antoniewicz

**Affiliations:** 1 Urology Department, Institute of Medical Sciences, Faculty of Medicine, Collegium Medicum, Cardinal Stefan Wyszyński University in Warsaw, Bursztynowa St. 2, 04-479 Warsaw, Poland; m.lipa@uksw.edu.pl (M.L.); a.antoniewicz@uksw.edu.pl (A.A.A.); 2 Department of Urology and Urological Oncology, Multidisciplinary Hospital in Warsaw-Miedzylesie, Bursztynowa 2 Street, 04-749 Warsaw, Poland

**Keywords:** Prostatic Neoplasms, Prostatic Neoplasms, Castration-Resistant, Neoplasm Metastasis, Lymph Node Excision, Salvage Therapy, Robotic Surgical Procedures, Lymphadenectomy, Ultrasonography, Interventional, Positron Emission Tomography Computed Tomography, Case Reports

## Abstract

**Background**: Metastatic castration-resistant prostate cancer (mCRPC) remains challenging due to progression despite androgen deprivation therapy (ADT). Current treatments, including androgen receptor-targeted agents, chemotherapy, bone-targeted agents, and PARP inhibitors, extend survival but face challenges, such as resistance, adverse effects, and limited durability. Metastasis-directed therapies (MDTs), such as stereotactic ablative radiotherapy (SABR), show promise in oligometastatic disease, but their role in oligoprogressive mCRPC is unclear. Salvage lymphadenectomy is rarely pursued due to invasiveness and limited data. This is the first report of robotic surgery as an MDT in this setting, demonstrating the potential of salvage robot-assisted video-endoscopic inguinal lymphadenectomy (RAVEIL) to manage oligoprogressive mCRPC and delay systemic progression. **Methods**: A 47-year-old male with metastatic hormone-sensitive prostate cancer (Gleason 10) underwent ADT, docetaxel chemotherapy, and radical retropubic prostatectomy with super-extended pelvic and retroperitoneal lymphadenectomy. Upon progression to oligoprogressive mCRPC, 68Ga-PSMA PET/CT detected a single metastatic inguinal lymph node. Salvage RAVEIL was performed using the da Vinci X™ Surgical System, guided by preoperative ultrasound mapping. **Results**: Histopathology confirmed metastasis in one of the eight excised lymph nodes. The patient achieved undetectable PSA levels and prolonged biochemical progression-free survival. Minor complications (lymphorrhea, cellulitis) resolved without sequelae. No further progression was observed for over 14 months. **Conclusions**: This case highlights RAVEIL as a viable MDT option for oligoprogressive mCRPC, potentially extending progression-free intervals while minimizing systemic treatment.

## 1. Introduction

Castration-resistant prostate cancer (CRPC) is an advanced stage of prostate cancer, characterized by disease progression despite castration levels of serum testosterone (≤50 ng/dL), as outlined in the 2024 European Association of Urology (EAU), the European Association of Nuclear Medicine (EANM), the European Society for Radiotherapy and Oncology (ESTRO), the European Society of Urogenital Radiology (ESUR), the International Society of Urological Pathology (ISUP), and the International Society of Geriatric Oncology (SIOG) guidelines on prostate cancer management. Patients with CRPC comprise a heterogeneous group: those without evidence of metastasis (nonmetastatic CRPC—nmCRPC) and those with metastatic disease (metastatic CRPC—mCRPC). mCRPC can be further divided into two categories: oligometastatic disease, in which metastases are limited in number and location, and polymetastatic disease, which is characterized by widespread metastatic involvement. A specific subset, oligoprogressive mCRPC, refers to cases in which disease progression occurs at a limited number of metastatic sites, presenting opportunities for localized treatment interventions.

Metastasis-directed therapy (MDT), including stereotactic ablative radiotherapy (SABR) and surgical metastasectomy, has demonstrated promise in the management of oligometastatic prostate cancer. According to the EAU–EANM–ESTRO–ESUR–ISUP–SIOG guidelines, MDT is considered a potential approach for patients with oligometastatic hormone-sensitive prostate cancer (mHSPC), particularly those with nodal recurrence (rcN1) detected on PSMA PET/CT. Retrospective studies suggest that MDTs, especially node-directed therapy, may improve cancer-specific survival (CSS), as demonstrated by an international study showing a significant survival benefit for patients receiving MDT compared to standard systemic therapy alone [1]. Furthermore, analyses indicate that elective nodal irradiation (ENRT) may reduce nodal recurrence rates compared to SABR, particularly in cases of pelvic lymph node recurrence [2]. However, despite promising results, the guidelines emphasize that MDT in patients with M1 remains investigational and should only be performed in highly selected cases within prospective studies or clinical trials.

Similarly, the American Urological Association guidelines acknowledge the potential role of MDTs in delaying systemic therapy in oligometastatic mHSPC but highlight the lack of high-quality prospective data demonstrating an overall survival (OS) benefit. Trials, such as STOMP [3] and ORIOLE [4], have shown that MDTs can prolong androgen deprivation therapy (ADT)-free survival and reduce disease progression compared to surveillance. However, the AUA guidelines emphasize that there is currently insufficient evidence to support MDT as a standard of care outside of clinical trials. Additionally, ongoing phase III trials like START-MET (NCT05209243) [5] and PRESTO (NCT04115007) [6] aim to further evaluate the impact of MDTs on long-term outcomes.

Notably, both EAU–EANM–ESTRO–ESUR–ISUP–SIOG and AUA guidelines highlight the lack of data regarding MDTs in metastatic castration-resistant prostate cancer (mCRPC). While retrospective studies suggest a potential benefit of MDTs in delaying systemic progression in select patients, no prospective trials have demonstrated a clear OS benefit in this setting. As a result, a surgical approach to MDT in mCRPC remains experimental, requiring further investigation before it can be integrated into routine clinical practice.

Here, we present the first report of robotic surgery used as an MDT in the context of oligoprogressive mCRPC, followed by a discussion synthesizing recent findings on MDTs in this clinical setting. This case highlights the potential of advanced imaging and localized therapies to improve outcomes while emphasizing gaps in current research.

## 2. Case Presentation

**Figure 1:**
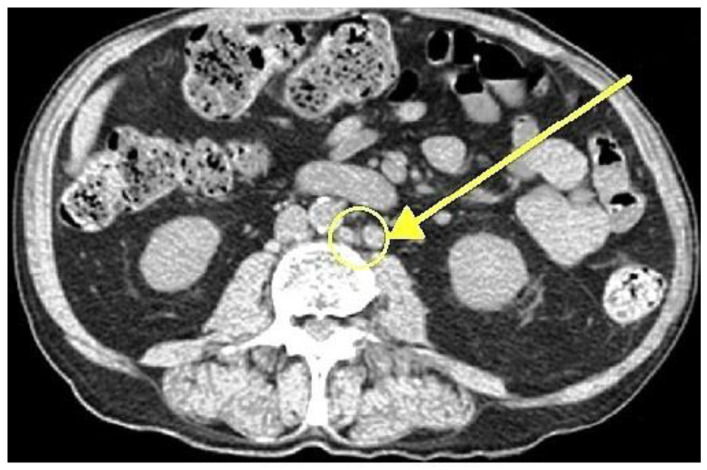
Axial contrast-enhanced CT scan demonstrating paraaortic lymphadenopathy (arrow indicating metastases of prostate cancer in lymph nodes).

In 2018, a 47-year-old male presented with a total PSA level exceeding 2100 ng/mL. Further diagnostic evaluation confirmed adenocarcinoma of the prostate, classified as ISUP Grade 5 Gleason score 10 (5 + 5) and staged using conventional imaging, including whole-body bone scintigraphy and whole-body computed tomography (CT), as cT2aN1M1a, indicative of newly diagnosed high-volume metastatic hormone-sensitive prostate cancer (per CHAARTED criteria). The patient reported no family history of prostate cancer or other malignancies. His medical history was significant for hypertension, which was well controlled with antihypertensive therapy. Initial conventional imaging revealed metastatic involvement of the obturator lymph nodes bilaterally, as well as the right external iliac, aortic bifurcation, paraaortic, and left renal hilum nodes, with a total of 14 affected sites (diameter > 8 mm in the pelvis and >1 cm in the abdomen, based on CT findings). Figure 1 illustrates the paraaortic lymphadenopathy.

### 2.1. Initial Management

The patient was treated initially with maximal androgen blockade (MAB), including degarelix combined with bicalutamide, a non-steroidal antiandrogen, followed by six cycles of early docetaxel chemotherapy. With a preoperative PSA level of 0.68 ng/mL, in October 2019, he was treated with a non-standardized, aggressive surgical approach comprising salvage radical retropubic prostatectomy (RRP), super-extended pelvic lymph node dissection (sePLND), and retroperitoneal lymph node dissection (RPLND). Intraoperatively, bilateral double-J ureteral stents were placed and later removed.

The surgical procedure lasted 230 min and included an intraoperative histopathological assessment of the bladder neck. The patient’s preoperative hemoglobin level was 7.9 mmol/L, which decreased slightly to 7.7 mmol/L postoperatively. The estimated intraoperative blood loss was 250 mL, and hospitalization lasted 10 days, with a surgical drain removed on postoperative day five due to persistent lymphorrhea.

A histopathological evaluation confirmed high-grade prostate adenocarcinoma with a Gleason score of 10 (5 + 5), demonstrating extensive bilateral infiltration, including seminal vesicles, with a pathological upstaging of pT3b. Surgical margins were negative. The resected specimens comprised the prostate with seminal vesicles, bladder neck (which was free of malignant infiltration), obturator lymph nodes bilaterally, external iliac lymph nodes bilaterally, paraaortic lymph nodes, and lymph nodes from the left renal hilum. Of the excised pelvic lymph nodes, one of five harbored micro-metastatic disease, while two larger nodes exhibited involutional degenerative alterations. Three additional nodes displayed fatty atrophy. Extensive fibrosis with multiple metastatic deposits was observed in the paraaortic lymph nodes and those located in the left renal hilum.

The patient achieved undetectable PSA levels postoperatively, with castration-level testosterone maintained through continued androgen deprivation therapy (ADT) comprising 45 mg of leuprolin acetate every six months.

### 2.2. Detection of Disease Progression

A rise in PSA to 0.020 ng/mL, despite castration-level testosterone, prompted restaging with a Ga-68-PSMA PET/CT scan on 11 March 2021. The scan detected radiotracer uptake in a single right inguinal lymph node with an SUVmax of 12.4, with no evidence of M1b (bone metastases) or M1c (visceral metastases) disease. The identification of this radiotracer-avid lesion, indicating new metastatic progression despite ongoing ADT, confirmed the diagnosis of oligoprogressive mCRPC. A physical examination revealed no palpable lymphadenopathy, with no other significant abnormalities detected. The diagnostic assessments were straightforward, with imaging confirming the diagnosis without significant challenges.

### 2.3. Preoperative Evaluation and Targeting

**Figure 2:**
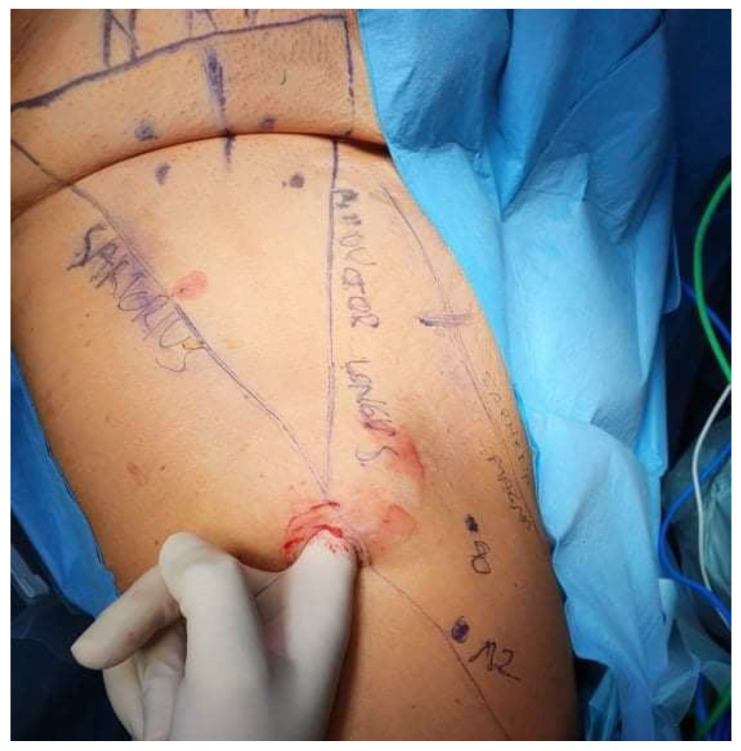
Detailed mapping of the targeted anatomy included the identification of key anatomical landmarks, such as the femoral nerve, femoral artery, femoral vein, great saphenous vein, the femoral triangle (sartorius muscle, adductor longus muscle, and inguinal ligament), and four suspicious lymph nodes.

Detailed mapping of the targeted anatomy was performed preoperatively (Figure 2). Ultrasound was utilized to identify key anatomical landmarks, including the femoral nerve, femoral artery, femoral vein, great saphenous vein, the femoral triangle (sartorius muscle, adductor longus muscle, and inguinal ligament), and four suspicious lymph nodes. These nodes were identified on ultrasound based on established malignancy criteria, including a round shape with a long-to-short axis ratio < 2:1, absent or displaced hilum, lower echogenicity than surrounding muscle tissue, and peripheral vascularity patterns observed on Doppler imaging. While the DROP-IN study demonstrated the advantages of PSMA PET/CT-guided intraoperative navigation with a robot-assisted DROP-IN gamma probe, achieving a 100% detection rate of PSMA-avid lesions, this technology was unavailable in our setting [7]. Preoperatively marked areas of lymph nodes identified as suspicious on ultrasound allowed for intraoperative control, ensuring that all targeted nodes were effectively removed.

### 2.4. Salvage Surgical Intervention

**Figure 3:**
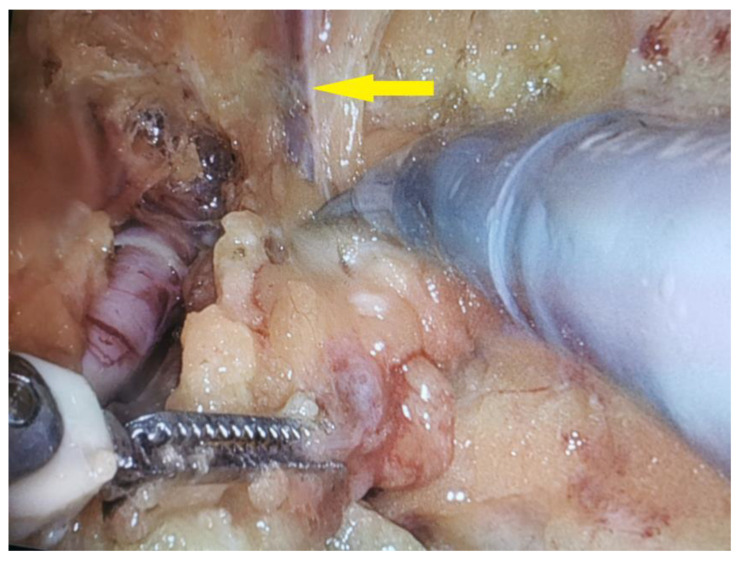
Intraoperative view of a robot-assisted video-endoscopic inguinal lymphadenectomy (RAVEIL) with the intention of preserving the great saphenous vein (indicated by the yellow arrow).

**Figure 4:**
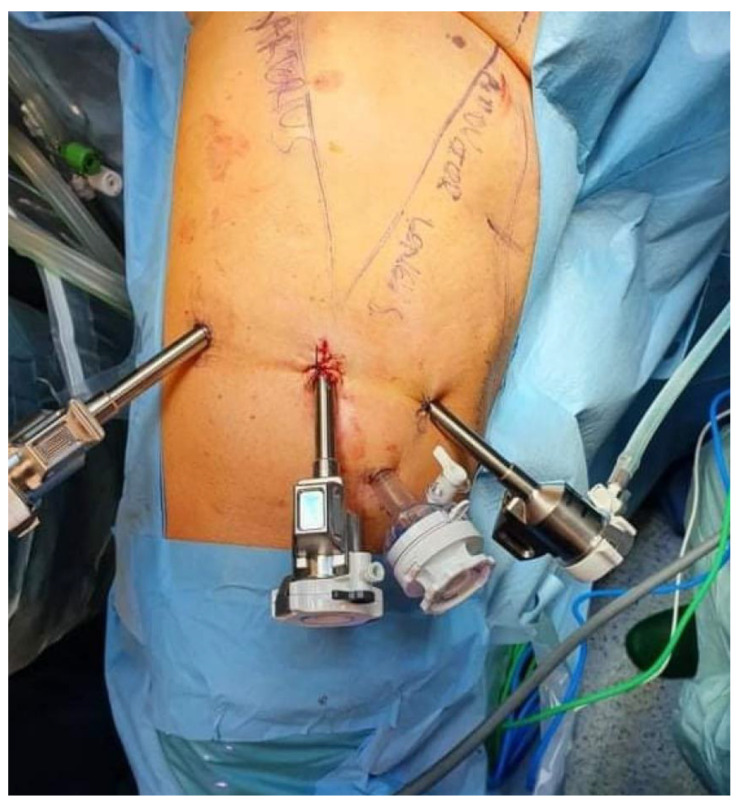
Scheme of trocar placement for robot-assisted video-endoscopic inguinal lymphadenectomy (RAVEIL).

On 12 August 2021, the patient underwent a right-sided salvage robot-assisted video-endoscopic inguinal lymphadenectomy (RAVEIL) without saphenectomy (Figure 3). The trocar docking scheme is shown in Figure 4. The procedure, performed using the da Vinci X™ Surgical System (Intuitive Surgical, Sunnyvale, CA, USA), lasted 50 min and was characterized by minimal blood loss and a hospital stay of three days (two days postoperatively). To prevent chylous drainage and lymphocele formation, two Redon drains were placed. The histopathological examination of the excised tissue included eight lymph nodes, with one node confirmed to have metastatic involvement.

### 2.5. Postoperative Course and Complications

The postoperative course was characterized by minor (Clavien–Dindo Grade II) complications, specifically readmission due to subcutaneous cellulitis, which was successfully managed with oral antibiotics (amoxicillin + clavulanic acid), and persistent chylous drainage (lymphorrhea) lasting over two weeks, requiring the prolonged use of a surgical drain. Despite these challenges, the patient recovered fully, with no further significant issues. The patient reported satisfaction with the treatment outcomes and noted no significant impact on quality of life (QoL), which was assessed using the Functional Assessment of Cancer Therapy-Prostate (FACT-P) questionnaire before the procedure and three months postoperatively. The initial FACT-P score was 130 out of a maximum of 156, indicating a good overall condition despite ongoing androgen deprivation therapy (ADT). Three months after the surgery, the FACT-P score increased slightly to 132, suggesting no negative impact on the patient’s quality of life and a potential improvement in some domains. The detailed subscale scores are presented in Table 1.

### 2.6. Follow-Up

**Figure 5:**
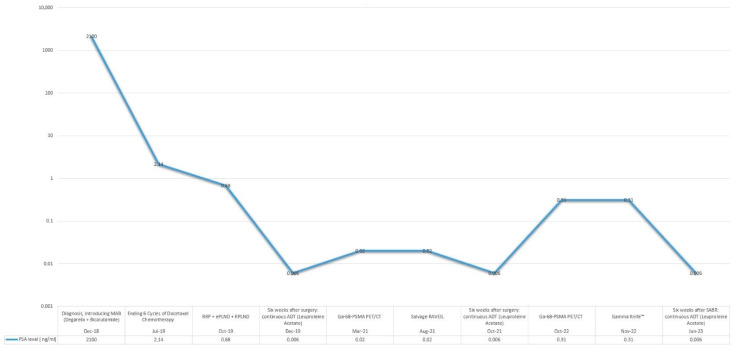
PSA response to sequential treatments over disease course.

Six weeks postoperatively, the patient demonstrated a PSA level that remained undetectable under continued ADT for 14 months. Adherence and tolerability were assessed through regular clinical follow-ups, with the patient reporting no major issues related to ongoing ADT. However, a subsequent rise in PSA to 0.31 ng/mL prompted periodic restaging with 68Ga-PSMA PET/CT, which identified recurrence localized to the region of the left renal hilum. The recurrence was treated with SABR using the Gamma Knife™ (by Elekta AB, Stockholm, Sweden), successfully reducing the PSA level to undetectable levels once again, with no further evidence of disease progression. Table 2 provides a summary of evidence supporting SABR in this setting. A summary graph depicting the overall course of the disease, including PSA dynamics and key treatment interventions, is shown in Figure 5.

## 3. Discussion

### 3.1. Clinical and Economic Justification of Using RAVEIL in Oligoprogressive mCRPC

#### 3.1.1. Rationale Behind Selection of Robot-Assisted Surgery

Although most studies on RAVEIL focus on penile cancer, the surgical technique is identical, involving the same dissection planes, steps of surgery, and robotic platform. A recent meta-analysis by Ge et al. demonstrated that RAVEIL achieved a significantly lower overall complication rate compared to open approaches (OR = 0.26, 95% CI: 0.09–0.70, *p* < 0.05). This suggests a major advantage in reducing surgical morbidity [18]. Additionally, a systematic review and meta-analysis by Patel et al. reported that robot-assisted approaches resulted in a 1.06-day reduction in hospital stay (mean difference: −1.06 days, 95% CI: −1.14 to −0.98, *p* < 0.001) and a 2.82-day reduction in the duration of drainage (mean difference: −2.82 days, 95% CI: −3.21 to −2.43, *p* < 0.001) compared to open procedures, highlighting their clinical benefits [19].

#### 3.1.2. Expected Benefits Compared to Conventional Surgery

Beyond reducing surgical morbidity, RAVEIL has demonstrated multiple advantages over conventional open inguinal lymphadenectomy (OILND), particularly in perioperative outcomes and complication rates. Findings from studies focused on penile cancer can be extrapolated per analogiam to support the potential advantages of RAVEIL in the management of oligoprogressive mCRPC. A meta-analysis by Patel et al. reported that robot-assisted approaches significantly lowered the rates of major complications (OR = 0.11, 95% CI: 0.05–0.24, *p* < 0.001) and reduced the incidence of skin necrosis (OR = 0.12, 95% CI: 0.05–0.28, *p* < 0.001) compared to OILND [19]. These findings are particularly relevant, given the high morbidity associated with open inguinal dissections in patients undergoing lymphadenectomy. A retrospective cohort study by Singh et al., which compared RAVEIL with OILND in 151 patients with penile cancer, reported a significantly lower incidence of major complications in the robotic group (2% vs. 17%, *p* = 0.0067), with no difference in lymph node yield (13 vs. 12.5 nodes, *p* = 0.44). Additionally, the robotic approach was associated with a significantly shorter hospital stay (median: 3 vs. 4 days, *p* = 0.0008) and reduced drainage duration (12 vs. 15 days, *p* < 0.0001) [20]. A systematic review by Zahid et al., which analyzed RAVEIL across multiple urologic oncology procedures, reported that the robotic approach led to significantly lower intraoperative complications (2.6% vs. 5.6% for the laparoscopic approach, *p* < 0.001) and reduced postoperative complications (24.53% vs. 32.03%, *p* = 0.004). Furthermore, robot-assisted surgery was associated with a slight reduction in estimated blood loss (50–100 mL), though statistical significance varied across studies [21]. Collectively, these findings reinforce the advantages of RAVEIL in minimizing perioperative complications while maintaining oncologic equivalence to open techniques, despite the limited data on this procedure in prostate cancer.

#### 3.1.3. Balancing Clinical Advantages and Economic Burden of Robotic Surgery in Highly Selected Cases of Oligoprogressive mCRPC

The economic feasibility of RAVEIL must be evaluated not only in comparison to open surgery but also against the long-term financial burden of systemic therapies in mCRPC. While robotic surgery requires a high initial investment, it may offer cost-saving potential by delaying the need for continuous systemic therapy and reducing complication-related expenses. Systemic treatments, such as enzalutamide, abiraterone, and olaparib, impose significant financial burdens over prolonged treatment durations. A cost-effectiveness analysis by Goudarzi et al. comparing enzalutamide and abiraterone found that enzalutamide had an incremental cost-effectiveness ratio (ICER) of USD 6260 per quality-adjusted life year (QALY), well below the USD 18,261 acceptability threshold. Over ten years, enzalutamide incurred a total cost of USD 17,541, while abiraterone was USD 16,408, but enzalutamide provided a superior QALY gain (1.02 vs. 0.84) [22]. A retrospective claims analysis by Ramaswamy et al. evaluating patients with chemotherapy-naïve mCRPC found that enzalutamide resulted in significantly lower per-patient per-month (PPPM) healthcare costs compared to abiraterone (USD 8085 vs. USD 9092, *p* = 0.0002), with prostate cancer-related PPPM costs of USD 6321 versus USD 7280 (*p* < 0.0001) [23]. Xu et al. further examined the cost-effectiveness of olaparib for BRCA-mutated mCRPC, reporting that, in China, the base case ICER for olaparib was CNY 392,727.87 per QALY, with incremental costs of CNY 93,673.23 and an incremental QALY of 0.23, indicating that it was not cost-effective within the Chinese healthcare system. However, in the United States, olaparib was considered cost-saving, with a USD 69,675.20 reduction in costs while still achieving a 0.23 QALY gain [24].

These findings underscore the high cumulative costs of systemic therapies in mCRPC, raising the question of whether a localized, metastasis-directed surgical approach, such as RAVEIL, could provide a cost-effective alternative in highly selected cases. The average cost of a robotic surgical system is estimated at USD 2 million per unit, with per-procedure disposable costs ranging from USD 3500 to USD 4000 [21]. However, when considering that systemic therapy for mCRPC costs approximately CAD 254,743 (USD ~ 187,000) per year for commercially insured patients and CAD 195,547 (USD ~ 143,000) per year for Medigap-insured patients in Canada, the financial burden of continuous pharmacologic treatment remains substantial and likely exceeds USD 100,000 annually in similarly structured healthcare systems [25]. Therefore, the upfront investment in robot-assisted surgery may be justified if it leads to reduced dependency on systemic treatments and lower long-term healthcare costs.

### 3.2. Evidence Supporting Surgical Interventions and Their Clinical Impact in CRPC

Although surgical interventions have received less attention, their potential in oligoprogressive mCRPC is notable, as surgical metastasectomy could complement SABR by addressing isolated metastatic lesions resistant to systemic therapies. Oligoprogressive mCRPC represents a critical challenge in advanced prostate cancer management, and MDTs, including both SABR and surgical interventions such as RAVEIL, are increasingly explored as strategies to delay next-line systemic treatment (NEST).

Soma et al. presented a case of repeat oligoprogressive mCRPC in which a solitary lung metastasis was successfully treated with thoracoscopic pulmonary metastasectomy. The patient achieved undetectable PSA levels for nine months after surgery, highlighting the potential efficacy of metastasectomy in selected patients [26].

Nozaki et al. further highlighted the potential of surgical interventions in oligoprogressive mCRPC by demonstrating the efficacy of lymphadenectomy guided by 11C-choline PET/CT imaging. Of 12 patients treated, 7 underwent salvage lymphadenectomy for progressive lymph node metastases, achieving a median post-surgery PSA reduction of 86% (range: 23–100%). The median PFS after lymphadenectomy was 8.5 months (range: 2.8–25.3 months), and the median time to initiation of systemic therapy was delayed to 9.3 months (range: 2.8–25.3 months). Notably, one patient achieved complete remission lasting 25.3 months, highlighting the potential durability of surgical outcomes in well-selected patients. These findings underscore the role of advanced imaging in patient selection and the ability of surgery to provide substantial disease control, even in cases resistant to systemic therapy [27].

The ongoing MEDCARE phase III trial builds upon the findings of the phase II study by rigorously evaluating the integration of surgical interventions, including metastasectomy, alongside other progression-directed therapies in oligoprogressive mCRPC. While the MEDCARE phase II trial primarily focused on the feasibility and efficacy of SABR, it also highlighted the potential role of surgical approaches, such as metastasectomy, in managing oligoprogressive disease. Although no patients underwent metastasectomy in phase II, the trial underscored the importance of addressing all progressive lesions to achieve optimal clinical outcomes, paving the way for further research [13]. The phase III trial, a randomized multicentric study with a planned enrollment of 246 patients stratified by the number and location of progressive lesions, aims to provide critical insights into the role of metastasectomy in enhancing disease control, improving quality of life (QoL), and delaying systemic therapies. With OS as the primary endpoint and secondary outcomes including NEST-FS, local control rates, and treatment-related toxicity, this study seeks to validate the utility of surgical strategies within multimodal treatment protocols, offering a comprehensive framework for progression-directed therapy in mCRPC [28].

Pelvic exenteration surgery (PES) has demonstrated substantial potential for symptom management in locally advanced and symptomatic CRPC. In a study involving 103 patients, PES achieved symptom-free survival in 93.2% of cases, with a median symptom-free duration of 27.9 months. Additionally, the one-year and three-year symptom-free survival rates were 89.2% and 64.1%, respectively. The procedure reduced critical symptoms, such as urinary obstruction (62.1% preoperatively to 1.6% postoperatively), gross hematuria (41.7% to 0%), and opioid analgesic use (22.3% to 2.9%). The median overall survival reached 33.6 months, with 92.2% survival at one year and 43.7% at three years. Grade 3 and 4 complications occurred in 11.6% and 8.1% of patients, respectively, underscoring the importance of careful patient selection [29]. These findings reinforce the complementary role of surgery in managing localized progression, particularly when systemic therapies fail, offering durable symptom control.

### 3.3. The Role of Multidisciplinary Teams in Managing Oligoprogressive mCRPC

The optimal management of oligoprogressive mCRPC requires a comprehensive multidisciplinary team (MuDT) involving urologists, oncologists, radiologists, nuclear medicine specialists, and imaging experts. Belda-Ferre et al. reported that 62.8% of advanced prostate cancer cases presented at MuDT meetings required the collaboration of at least two specialties, emphasizing the complexity of care for these patients. Among surveyed physicians, 90.7% agreed that MuDTs improved clinical decision-making and patient satisfaction. However, only 60.5% of decisions made during MuDT meetings were binding, with variability in adherence across institutions, highlighting areas for improvement in integration and execution. Training in MuDT practices was found to be limited, with only 46.5% of respondents considering themselves adequately trained, although 37.2% reported that MuDT meetings significantly improved decision-making efficiency [30].

Zhu et al. demonstrated that dynamic MuDT discussions at key treatment milestones for metastatic CRPC significantly improved OS. Patients participating in MuDT meetings had a median OS of 39.7 months compared to 27.0 months for non-participants (HR: 0.542, *p* < 0.001). This survival benefit persisted across subgroups, including those undergoing first-line therapy (median OS: not reached vs. 27.0 months, *p* < 0.001) and multi-line therapy (median OS: 36.7 months vs. 25.6 months, *p* = 0.044). Moreover, MuDT involvement correlated with higher rates of advanced diagnostic utilization, with 38.4% of MuDT patients receiving multi-line therapies compared to 21.8% of non-MuDT participants (*p* < 0.001), underscoring the importance of MuDTs in coordinating complex treatment strategies [31].

### 3.4. Limitations and Future Directions

The surgical management of oligoprogressive mCRPC as a modality of MDT faces several critical limitations. First, as mentioned above, the existing evidence base for surgical interventions is significantly weaker than for SABR. While SABR is supported by prospective trials demonstrating consistent outcomes in delaying disease progression, most surgical studies are retrospective or based on small, single-center cohorts, which limits their reliability and generalizability. Additionally, robot-assisted surgical techniques, such as RAVEIL, though promising, lack robust comparative data against SABR or other MDT modalities. However, as demonstrated in this case report, RAVEIL shows potential for advancing the surgical management of oligoprogressive mCRPC by achieving excellent local control, prolonged biochemical progression-free survival, and minimal complications. These findings highlight the need for further studies to validate its efficacy and establish its role within MDT strategies. A further limitation lies in the higher perioperative risks and resource requirements associated with surgery. Unlike SABR, which is non-invasive and widely accessible, surgical approaches require specialized expertise, advanced equipment, and longer recovery times. These logistical and clinical challenges hinder the broader adoption and integration of surgery into standard MDT protocols for CRPC. Finally, the lack of standardization in reporting outcomes for surgical interventions complicates comparisons across studies. Definitions of key concepts, such as “oligoprogression”, and the reporting of endpoints, such as PFS or systemic treatment-free survival (STFS), vary widely, making meta-analyses and evidence synthesis difficult. This case report underscores the importance of detailed and standardized outcome reporting in surgical studies, providing a valuable foundation for future research.

To address these gaps, future research should prioritize the development of high-quality, prospective randomized controlled trials (RCTs) directly comparing surgical interventions with SABR in oligoprogressive mCRPC. These trials should emphasize robust endpoints, such as PFS, OS, QoL, and systemic treatment-free intervals, while also incorporating cost-effectiveness analyses to evaluate broader implications. Advanced imaging techniques, such as PSMA PET/CT, should be systematically integrated into surgical research to optimize patient selection and enhance procedural precision. The use of PSMA PET/CT for precise patient selection and the identification of oligometastatic lesions has been shown to improve the effectiveness of MDT, leading to prolonged progression-free survival compared to earlier imaging modalities, such as choline PET/CT [32]. Prospective studies should assess how imaging-guided approaches can improve outcomes by identifying metastatic lesions that may benefit more from surgical resection than from SABR. Collaborative multicenter studies will be essential in overcoming challenges associated with the rarity of oligoprogressive mCRPC. International networks pooling resources and expertise can enable larger patient cohorts and more statistically robust analyses. Additionally, biomarker-driven research should identify specific patient subgroups most likely to benefit from surgical interventions, facilitating personalized treatment approaches. Finally, the integration of surgery into multimodal treatment regimens offers potential for enhanced outcomes. Combining surgery with systemic therapies, such as androgen receptor inhibitors (ARIs), immunotherapies, or radiopharmaceuticals like 177Lu-PSMA-617, could provide synergistic effects and improve long-term disease control. Future research should also investigate the optimal timing of surgical interventions within the treatment continuum, particularly in relation to systemic therapies and radiotherapy.

## 4. Conclusions

Building on the evidence supporting MDTs in oligoprogressive mCRPC, salvage RAVEIL emerges as a promising surgical approach within this therapeutic framework. By utilizing advanced imaging modalities, such as 68Ga-PSMA PET/CT for precise targeting, this approach enables localized treatment while reducing reliance on systemic therapies. In our case report, RAVEIL demonstrated durable disease control with minimal morbidity, emphasizing its practicality and safety in clinical practice.

These findings support the inclusion of RAVEIL in a multidisciplinary framework for mCRPC management, particularly for patients with isolated metastatic progression. Future prospective studies are needed to confirm its efficacy and long-term outcomes compared to non-invasive MDT modalities, such as SABR. Moreover, expanding the application of robot-assisted surgical techniques to other metastatic sites could further enhance their role in MDT strategies for mCRPC. Enhancing collaboration among specialties and further advancements in imaging and surgical techniques will be essential for optimizing RAVEIL and other robot-assisted approaches in personalized oncology care.

## Figures and Tables

**Table 1:** FACT-P subscale scores.

Subscale	Maximum Score	Preoperative Score	Postoperative Score
Physical Well-Being (PWB)	28	22	22
Social/Family Well-Being (SWB)	28	26	27
Emotional Well-Being (EWB)	24	20	21
Functional Well-Being (FWB)	28	24	25
Prostate Cancer Subscale (PCS)	48	38	37

**Table 2:** Summary of clinical trials evaluating SABR in oligoprogressive mCRPC.

Trial	Intervention	Findings	Key Clinical Endpoints
Kwan et al. (2021) ICE-PAC [8]	Stereotactic Ablative Body Radiotherapy (SABR) + Immune Checkpoint Inhibitor (ICI) (avelumab)	48% disease control rate; ORR 31%; systemic benefit noted; safety—90% Treatment-Related Adverse Events (TRAEs); 16% grade 3–4; 10% discontinued treatment	Radiographic progression-free survival (rPFS)—8.4 months; overall survival (OS)—14.1 months.
Zhang et al. (2021) [9]	SABR; SABR + Poly (ADP-ribose) Polymerase (PARP) inhibitors	39.5% progression-free at 1 year; 20.8% at 2 years; delayed systemic progression; sustained local control; patients with BRCA1/2 mutations had improved progression-free survival (PFS) with SABR + PARP inhibitors	Prostate-Specific Antigen (PSA) PFS—9.2 months; local PFS—84.4% at 1 year, 75.3% at 2 years; Distant Metastasis-Free Survival (DMFS)—17.6% at 1 year, 5.0% at 2 years; BRCA1/2 PFS—13.2 months (vs. 6.8 for PARP inhibitors alone); time to systemic progression—16.4 months (vs. 9.1 for systemic therapy (ST), *p* < 0.01).
Onal et al. (2021) [10]	SABR	73.1% PSA response; 48.6% oligometastatic relapse; 35.1% diffuse progression; effective in most cases	OS (2-year)—86.9%; PFS—16.6 months; Time to Next Systemic Therapy (TTNST)—16.4 months.
Deek et al. (2021) [11]	SABR; SABR + Docetaxel	SABR significantly delayed PSA failure, Time to Next Intervention (TTNI), and DMFS; 2-year OS improved vs. systemic therapy (ST) alone; SABR + Docetaxel showed an OS benefit over Docetaxel monotherapy, particularly in low-volume metastatic disease	PSA Failure—9.7 months; TTNI—14.9 months; DMFS—12.7 months, 2-year OS—90.3%; SABR + Docetaxel OS—42.6 months (vs. 29.8 months for Docetaxel alone, Hazard Ratio (HR)—0.65, *p* = 0.008).
Pan et al. (2022) [12]	Stereotactic Body Radiotherapy (SBRT) guided by dual-tracer Positron Emission Tomography/Computed Tomography (PET/CT)	SBRT improved Metastasis-Free Survival (MFS) compared to androgen deprivation therapy (ADT) alone; PSA response > 90% was achieved by 86% of patients with SBRT; minimal toxicity—no grade ≥ 3 Adverse Events (AEs)	MFS—11.0 months (ADT) vs. not reached (SBRT); HR—4.69 (95% Confidence Interval (CI): 2.92–25.0, *p* < 0.001); PSA response > 90% in 86% of patients with SBRT.
Rans et al. (2024) MEDCARE [13]	SABR	Delayed systemic progression; significantly prolonged Next-Line Systemic Treatment-Free Survival (NEST-FS) (31 vs. 13 months in treated vs. untreated lesions); 2-year local control rate 95%	NEST-FS—17 months; Local Control—95%.
Francolini et al. (2024) ARTO [14,15]	SABR + Abiraterone	Enhanced efficacy vs. systemic therapy (ST) alone; improved disease control; OS benefit observed but not statistically significant (*p* = 0.07)	PFS—10 months; OS—114 months.
Le Guevelou et al. (2024) [16]	SABR + Androgen Receptor Inhibitors (ARIs); SABR + Lutetium-177 Prostate-Specific Membrane Antigen (177Lu-PSMA-617)	Extended PFS (HR: 0.35); low toxicity (grade 3 < 5%); prolonged systemic treatment-free survival; SABR + 177Lu-PSMA-617 improved biochemical PFS and reduced skeletal-related events	PFS extended (HR: 0.35); NEST-FS—2–3 years; SABR + 177Lu-PSMA-617: biochemical PFS—12.1 months (vs. 7.4 for 177Lu-PSMA-617 alone, *p* = 0.045); skeletal-related events—10% (vs. 22% for radiopharmaceutical-only group).
Eule et al. (2024) [17]	SABR; SABR + Next-Generation ARIs	Delayed need for systemic therapy; 40.6% PSA response; treating all lesions further improved TTNST; extended treatment efficacy; SABR + ARIs prolonged Time to Progression (TTP) and enhanced PSA response	TTNST—10.1 months; OS—40.3 months; SABR + ARIs: TTP—14.5 months (vs. 8.3 months for ARIs alone, *p* = 0.03); PSA reduction ≥ 50% in 35% of cases within six months.

## Data Availability

All data underlying the findings of this study are available within this publication. Patient data from the Multidisciplinary Hospital in Warsaw-Miedzylesie have been anonymized to ensure confidentiality. Due to ethical and legal restrictions related to data protection regulations, raw patient data cannot be shared publicly. Requests for further information may be directed to the corresponding author.
